# Release of nifedipine, furosemide, and niclosamide drugs from the biocompatible poly(HEMA) hydrogel structures

**DOI:** 10.55730/1300-0527.3474

**Published:** 2022-09-01

**Authors:** Bahar YILMAZ

**Affiliations:** Department of Bioengineering, Karamanoğlu Mehmetbey University, Karaman, Turkey

**Keywords:** Hydrogel, drug release, cytotoxicity, furosemide, nifedipine, niclosamide

## Abstract

The primary aim of this article was to improve the solubility and bioavailability of the drugs nifedipine, niclosamide, and furosemide due to their poor solubility and dissolution rate. Therefore, these drugs require improvement in solubility and dissolution rate in formulation development, especially in solid dosage forms such as hydrogels and capsules. Hydrogel structures were synthesized by using a biocompatible, nontoxic, and protective pure 2-hydroxy ethyl methacrylate (HEMA) monomer. These hydrogel structures were characterized by various techniques such as scanning electron microscopy and Fourier-transformed infrared spectroscopy. In the next step, drug molecules were loaded in the poly (HEMA) hydrogels. Drug releases of drug-loaded hydrogel structures were measured at certain time intervals and recorded cumulatively (%). The pH affinity, morphology, structure, drug release, swelling, and cytotoxic effect of the resulting materials were studied in detail. In addition to investigating the biocompatibility and cytotoxicity of the poly (HEMA) hydrogel, we evaluated an in vitro cytotoxicity assay on MCF-7and MIA PaCa-2 and HEK 293 cell lines with the hydrogel structure and confirmed cell viability of over 85%. These results suggest that our HEMA polymeric hydrogel is a material with biological importance and great pharmacological potential as a supporting material in the drug release of nifedipine, furosemide, niclosamide, and similar drug molecules.

## 1. Introduction

Recently, different controlled drug delivery applications for the treatment of diseases have become available in modern medical technology [1]. Choosing the appropriate route of application affects how easily a drug reaches the target area, drug dosage, and dosage regimen [2]. It may thus be highly beneficial to monitor the release behavior of the drug delivery platform [3]. This platform can be useful for the continuous treatment or for targeted, site-specific drug release [4].

One potential way to gain control over the release behavior is the application of a smart polymer coating [5]. The smart polymeric coating can serve several purposes simultaneously in such systems. Polymeric encapsulation, which restricts environmental exposure, may achieve stabilization of the solid state of a drug [6]. Besides, these coatings may be helpful for several therapies as they make possible controlled drug release and reduce serious side effects [7]. The main functions of drug delivery systems are based on the mechanism of safely moving the drug to various parts of the body, controlling the amount of medication [8–9], and duration of treatment [10–11]. In drug release, most of the characteristics of a carrier material are required, such as biocompatibility, nontoxic immunogenicity deficiency, repeatability, and continuous activation to the target [12]. Stimulant-sensitive hydrogels that show great potential in a variety of pharmaceutical and biomedical applications have been one of the latest advances in carrier materials science [13].

Hydrogels have various uses in biomedical applications [14]. At the same time, they are well-suited for regenerative medicine and controlled drug delivery [15]. Hydrogels are networks of hydrophilic homopolymers or copolymers that inflate water and these are biological fluids that are absorbed [16]. Due to these characteristic features, hydrogels offer important opportunities for drug delivery systems [17]. In particular, hydrogel structures can cause a slow decomposition and release of a water-insoluble drug. They can also protect the drug from stomach acid and enzyme corrosion, thus improving the stability of the drug [18–19]. Hydrogels improve the release behavior, degradation, and stability of drugs, such as furosemide, niclosamide, and nifedipine by maintaining drug concentration and overcoming biological barriers to cellular uptake in a therapeutic study [19–20].

The drugs niclosamide, furosemide, and nifedipine are water-insoluble, antiinflammatory drugs used to relieve cardiovascular disease or inflammation [20–21]. Nifedipine is an effective calcium channel blocker widely used in various cardiovascular diseases for clinical management [22]. In a number of cases, furosemide is a loop diuretic that is used orally to change the amount and/or composition of body fluids, including hypertension heart failure, renal failure, nephrotic syndrome, and cirrhosis [22–24]. Niclosamide has been recognized as an anthelminthic drug almost since it was known. Moreover, it is an active medication against most tapeworms [25]. Niclosamide is also used as a molluscicide for sewage treatment in schistosomiasis prevention programs [26]. However, the emergence of the anticancer properties of niclosamide has been in the recent past. Recently, there has been a notable increase in studies showing the anticancer effect of the drug niclosamide against many cancer diseases, such as the ovary, prostate, colon, and breast cancer [27–28]. Recent studies show that hydrogel structures and/or nanofiber structures increase the preservation of these drugs by forming host-guest complexes [29–30]. At the same time, the use of hydrogels as a unifying matrix prevents the degradation of nifedipine, niclosamide, and furosemide [28]. Therefore, the main functions of hydrogels are to prevent drug release behavior and drug degradation in drug delivery applications.

In the field of drug delivery, there are many studies on combining polymeric structures such as hydrogels and films [31–32]. However, there are only a limited number of studies performed on the inclusion of low-resolution active agents such as anticancer drugs, antibacterials, essential oils, and aromas into hydrogel [33–34]. Hydrogels can exhibit the same mechanical properties (water content, biocompatibility, transport mechanism, etc.) as soft tissues [35]. They can therefore be used as physical models for solid tumors or integrated into drug and biomolecule release studies [36–37]. HEMA hydrogel was used as supporting material for rapid in vitro assessment of three drug release profiles: niclosamide, nifedipine, and furosemide [38–39]. These drug molecules are generally used to support cancer drugs. These drug molecules with low solubility were loaded onto the HEMA hydrogel to improve bioavailability and protection [40–41]. The comparative cytotoxic effects of these drug molecules were studied for the first time in this study. Thus, it was determined that the drug molecules showed a more cytotoxic effect thanks to the HEMA hydrogel.

The objective of this study was to examine the release of low water solubility drug molecules from hydrogel (furosemide, nifedipine, and niclosamide) and their effect on cancer cells. Therefore, synthetic, biodegradable, and HEMA-based hydrogels were created. The preservation of furosemide, nifedipine, and niclosamide structures was improved by complexing with the hydrogel structure of HEMA. Then, the release of drug molecules from the hydrogel structure was enabled. Cytotoxicity tests of the released drug molecules against MCF-7, HEK 293, and MIA PaCa-2 cell lines were performed. The results showed that HEMA hydrogel structure can hold great promise in promoting the preservation and release of drugs into cells.

## 2. Materials and methods

### 2.1. Materials

2-Hydroxyethyl methacrylate, ≥99%, (HEMA), MBA (N-N′ methylenebisacrylamide), APS (Ammonium persulfate), and TEMED (N,N,N′,N′-Tetramethyl ethylenediamine), drug molecules (nifedipine, niclosamide, and furosemide) were obtained from Sigma and Merck. The phosphate buffer solution, the HEMA hydrogel, and drug solutions were prepared in our laboratory. Besides, breast carcinoma line MCF-7 (ATCC ® HTB-22 ™), pancreatic carcinoma line MIA PaCa-2 (ACC 733, DSMZ), and epithelial HEK-293 (ATCC ® CRL-1573 ™) lines were purchased from Sigma.

### 2.2. Synthesis of HEMA hydrogels

Poly (HEMA) hydrogels were synthesized by the bulk polymerization method. This method involves only the HEMA monomer molecule, an initiator APS (Ammonium persulfate), and crosslinker agents such as MBA (N-N′ methylenebisacrylamide) and TEMED (N,N,N′,N′-Tetramethyl ethylenediamine). The monomer is taken in the liquid state and the initiator is dissolved in the monomer [28]. In this way, the monomer mixture was prepared. HEMA (0.137 mL) and other components of APS (0.002 g), MBA (0.0077 g), and TEMED (10 μL) were dissolved/dispersed and then they were mixed at room temperature under magnetic stirring at 400 rpm. After complete dissolution, the mixtures were injected into molds of glass plates. The molds were transferred to an environment where polymerization was carried out at +4 °C for 12 h and then at −20°C for 24 h [28].

### 2.3. Drug loading

Prepared hydrogels were weighed and immersed in PBS solution (4 mL, 0.01 M, pH 7.0) containing 0.2 mg/mL drug. The hydrogels were incubated at room temperature without shaking and samples (100 μL) of the drug loading solution were collected at predetermined time points for up to 24 h. The amount of loading furosemide, nifedipine, and niclosamide drugs in hydrogel samples was determined using an UV–Vis spectrophotometer at 513, 440, and 340 nm, respectively. The drug concentration was determined by spectrophotometric measurements, and the amount of drug loaded on the hydrogels was calculated from the difference between the initial and final drug amount in the loading solution [15] (Schema).

### 2.4. FT-IR analysis

The structure of 2-Hydroxyethyl methacrylate (HEMA) hydrogels and drug-loaded HEMA hydrogels were characterized by performing FT-IR spectroscopy. Fourier transform infrared attenuated total reflectance spectra were recorded using FT-IR spectrophotometer (Bruker Vertex 70 ATR-FTIR) over the range 4000–400 cm^−1^.

### 2.5. Morphology

The morphology and structure of the HEMA hydrogels were examined by scanning electron microscopy (SEM). Hydrogel samples were coated with 5 nm Au prior to SEM imaging. The SEM images of hydrogels were obtained at a distance of approximately 1 μm.

### 2.6. Swelling measurement

Equilibrium swelling (ES) of free hydrogels was determined in distilled water and different buffer solutions. HEMA hydrogels have been immersed into 50 mL of prepared solution at 25 °C for 18h to reach maximum swelling capacity.


$$\text{Equilibrium swelling }\%\;(\text{ES})=({\text{W}}_{2}-{\text{W}}_{1})/{\text{W}}_{1}\times 100$$

where W_1_ is the initial dried sample weight, and W_2_ is the sample weight after 18 h swelling [16]. The swelling ability of the hydrogels was also determined in different buffers (pH 2, pH 4, pH 7, and pH 9). All these experiments were repeated three and/or four times.

### 2.7. In vitro drug release of HEMA

The drug release amount of drug-loaded HEMAs was determined hourly by the UV spectrophotometer in a predetermined buffer solution (Shimadzu UV 1800). All the measurements were completed in 96 h. All results were performed in triples and presented in the unit of cumulative release as seen in the following equation [23].


Cumulative release=Mt/M0×100

where M0 is the amount of drug preloaded into the hydrogel and Mt is the amount of drug released.

### 2.8. Cell culture and viability assay

Cell viability assessment was performed on MCF-7 (human breast adenocarcinoma), MIA PaCa-2 (human pancreatic carcinoma), and HEK 293 (human embryonic kidney 293) cell lines by using Alamar blue method. This method is based on color changes during the reduction of Alamar blue. It is also a common method used to test cell proliferation, viability, and/or cytotoxicity. Ninety-six-well plates containing pure HEMA and drug-loaded HEMA were prepared. The cells were seeded into these 96-well plates at a density of 10,000 cells/well. Each cell of the well containing 100 μL DMEM medium was incubated at 37 °C with 5% CO2 for 72 h. Next, 10 μL of Alamar reagent (per well) was added to each well and incubated at 37 °C, 5% CO2 for 4 h. After incubation, the absorbance of each well was measured at 560–600 nm. Following the incubation, the absorbance of each well was measured at 560–600 nm by using an iMark Microplate Reader (Bio-Rad) [30].

### 2.9. Detection of cytotoxicity in artificial blood samples

Cancer cells containing artificial blood (RBCs of expired human blood schema) were used for cytotoxicity studies. Once cancer cells with artificial blood were cultured at 80 μg/mL in these 96-well plates, drug molecules were added. Cell survival was observed by using the standard Alamar blue assay. Following 24-h incubation, the cultured medium was removed and replaced. Alamar blue in PBS was added and incubated at 37 °C for 4h. After incubation, the absorbance of each well was measured at 560–600 nm by using an iMark Microplate Reader (Bio-Rad) [42–43].

## 3. Results

**Figure 1:**
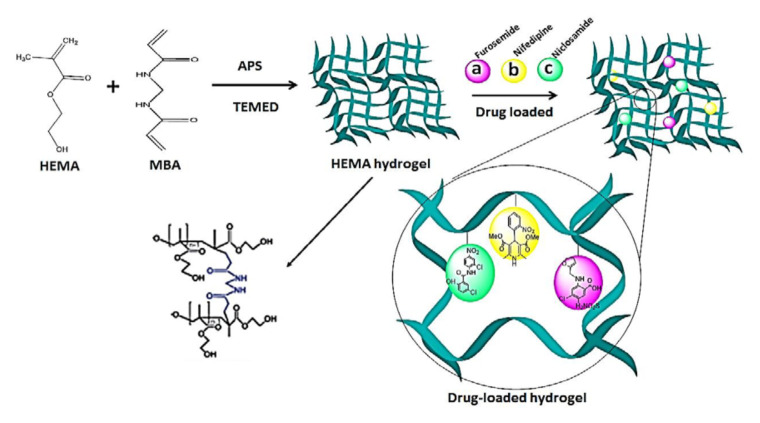
The schematic of the preparation process of HEMA hydrogel and drug-loaded HEMA hydrogel, (a) Furosemide, (b) Nifedipine, (c) Niclosamide.

The production of hydrogel delivery systems should preserve the bioactivity of the drug. Both the HEMA hydrogel and the drug (nifedipine, niclosamide, and furosemide) must be chemically and physically stable during packaging, transportation, and storage processes. HEMA-based hydrogels are structures that usually belong to the group of swelling, drug release, and controlled drug delivery systems. The preparation process of HEMA-based hydrogels is presented in Figure 1. In this work, bulk polymerization was used. The reaction between APS and TEMED was the first step of polymerization: Homolytic cleavage of APS fragments accelerated by TEMED created free radicals. We successfully synthesized HEMA-based hydrogel. The prepared HEMA hydrogel formed a complex with drug molecules that have low water solubility (Figure 1). The binding between the drug and HEMA was provided by noncovalent interactions, such as π-π, CH-π, electrostatic interaction, H-binding, and hydrophobic interaction [34–35]. When the drug is released, the hydrogel should be planned and designed either to degrade to avoid surgical removal or to be reused by drug refilling. These opportunities provided by HEMA are most likely due to the inclusion of the nonpolar parts of these molecules in the nonpolar spaces of HEMA [36]. The interaction between hydrogel and drug molecules decays in the presence of suitable environments. This is because our hydrogel structures swell only in the presence of suitable environments. The bulging hydrogel structures realize the release of drug molecules.

**Figure 2:**
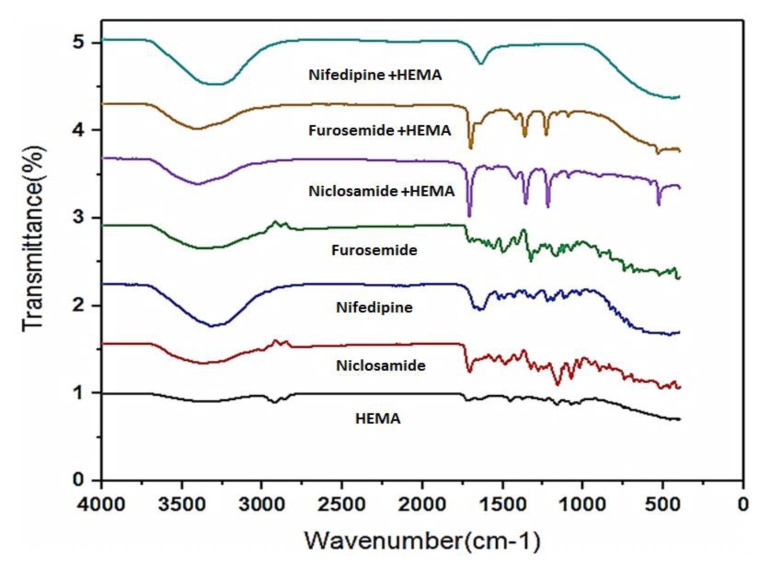
FT-IR spectra of pure HEMA hydrogels and drug-loaded hydrogels in the range 4000–400 cm^−1^.

The drug molecules have been successfully complexed with HEMA hydrogels to increase their preservation. Structural characterization was carried out using FTIR to detect the functional groups on the surfaces of drug molecules with different properties and the HEMA-based hydrogel structure and to determine their complex structures. At the same time, both pure HEMA structures and interactions between HEMA and drug molecules were shown. Characteristic imine (-N=CH) stretching vibration of hydrogel and drug molecules was seen at around 1600 cm^−1^. Broadband around 3300 cm^−1^ in the spectra of hydrogel and drug molecules was attributed to the stretching vibration of NH or OH (Figure 2). The observed peak that is located at 1642 cm^−1^ is the most significant difference between the loaded hydrogel samples (Figure 2). The peak at 1642 cm^−1^ occurred from the conjugated C = O bond. Due to C-O stretching, the peaks at 1070 cm^−1^ also confirmed the existence of the ester group of HEMA. The peak observed at 1154 cm^−1^ can be explained by the C-N stretching vibrations of the HEMA hydrogel containing the drug molecules of niclosamide and furosemide [37]. There is a significant increase in band intensities of C = O, C-H, C = C, and C-O stretching vibrations in drug-loaded hydrogel structures compared to pure drug structures.

**Figure 3:**
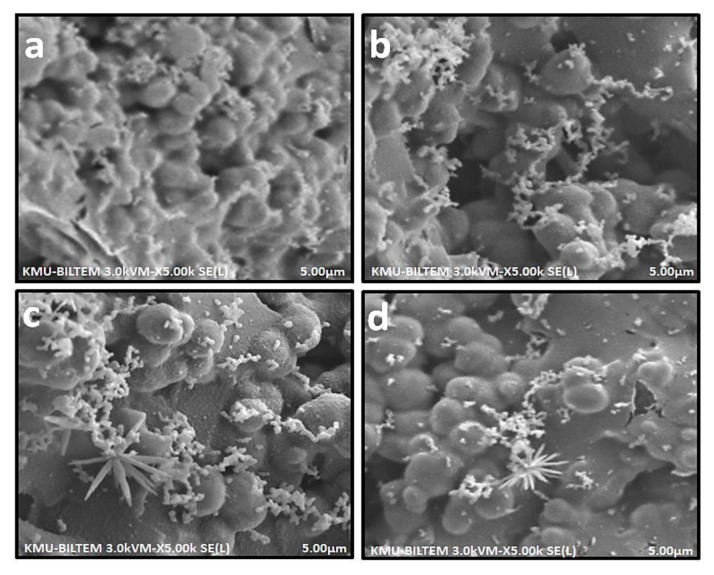
SEM images in cell presence of pure hydrogels and hydrogels containing drug molecule, (a) pure HEMA, (b) nifedipine loaded HEMA, (c) niclosamide loaded HEMA, (d) furosemide loaded HEMA.

The complexation of furosemide, niclosamide, and nifedipine with HEMA hydrogel was visualized with SEM after FT-IR characterization. The morphological properties of pure hydrogel (Figure 3a) and HEMA hydrogels containing drug molecules (Figures 3b–3d) were evaluated using SEM. Hydrogels were clearly observed to have porous internal structures and interconnected pores. The structure of pure hydrogels has been monitored to contain smoother structures compared to the drug-loaded hydrogels. The morphologies of hydrogels were observed in cell-containing mediums and images were compared. As drug releases occurred from the drug-loaded hydrogel in the cell environment, crystal structures were observed on the surface of the hydrogel. Since these crystal-like structures were not observed in the pure hydrogel, these structures can be considered drug molecules. This image indicated that the hydrogel structure protects and releases the drug molecules in the presence of cells. As shown in the images, all hydrogel structures clearly displayed the molecules they contained following the application of the process.

**Figure 4:**
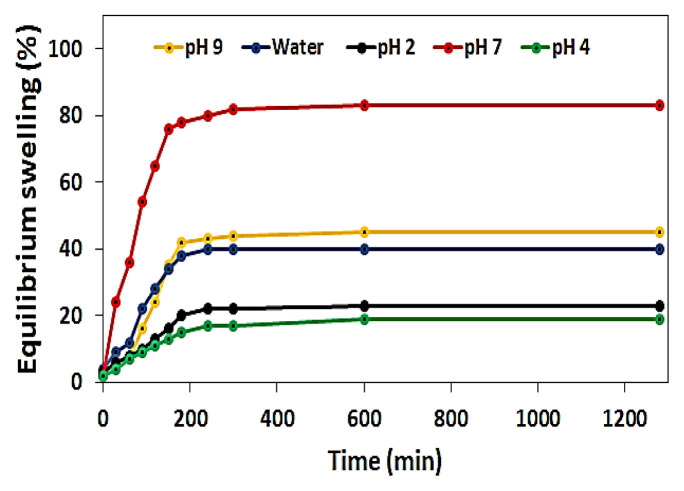
Equilibrium swelling behavior of hydrogels.

The equilibrium swelling behaviors of the HEMA hydrogels in different buffer solutions (pH 2, pH 4, pH 7, and pH 9) and water are shown in Figure 4. According to the swelling of HEMA, buffer solution was absorbed more than water. As the pH was increased, the swelling ability of the hydrogel increased. When the pH exceeds 7, the swelling of the HEMA structure was influenced negatively. The rising pH of solutions showed that the enlarging of hydrogel speed and size also increased. The increasing pH causes ionization of the carboxyl groups of MBA. Moreover, this causes the separation of hydrogen bonds between the carboxylic acid groups of the MBA and the oxygens of the ether groups of the HEMA. Combined with the electrostatic repulsion force, the dissociation of hydrogen bonds makes the hydrogel network swell rapidly [38–39]. The fewer hydrogen ions in the body, the better our overall health. The pH of our blood is in the range of 7.35–7.45 [40]. According to these values, we can say that the pH of our blood is alkaline. In healthy individuals, intragastric pH ranges from 1.3 to 2.5 in case of hunger, while during eating, pH can reach 7.5. An increase in the pH of the stomach with the introduction of food into the stomach triggers the secretion of gastric fluid [39–40]. Drug molecules or active substances are usually taken into the body in satiety. In the case of satiety, the body pH is usually around 7–7.5. Since the optimal working pH of HEMA hydrogel is around 7, its biological properties do not have any problems.

**Figure 5:**
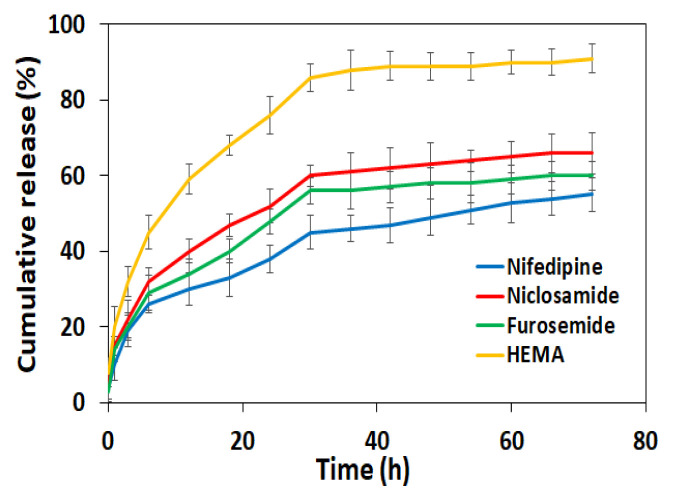
Cumulative drug release in HEMA hydrogel as a function of time. The results are expressed as a mean ± standard deviation (n = 3).

The rapid drug release from the hydrogel structure was observed in the first hours (Figure 5). Thereafter, a gradually decreasing rate of release was observed up to 20 h after which the release remained relatively constant. The drugs were not completely released within 48 h. The percentage of loaded drug amounts released in 48 hours was 68.9 ± 4.7%, 65.2 ± 2.3%, and 71.3 ± 5.1% for niclosamide, nifedipine, and furosemide, respectively.

Structural features and monomer types of hydrogels are very significant to decide the application field. For instance, evaluation of the structural properties of hydrogel structures is very important in various biological fields such as drug delivery, biomedical applications, and tissue engineering. As seen in Table 1, hydrogel structures were used in many areas, especially drug release. In addition, drug release, solubility, and vasoactivity studies were carried out from some hydrogel structures of nifedipine, niclosamide, and furosemide drug molecules. In this study, HEMA-based hydrogel structures containing drug molecules have been designed for cancer therapy. It has been observed that this hydrogel structure swells when contacted with an aqueous solution and/or pH solution. At the same time, this hydrogel structure releases the drug molecules it contains by providing the ability to swell in an aqueous environment. In this section, we shall discuss the swelling of hydrogels for cancer treatment and the release of each drug in the HEMA hydrogel.

**Figure 6:**
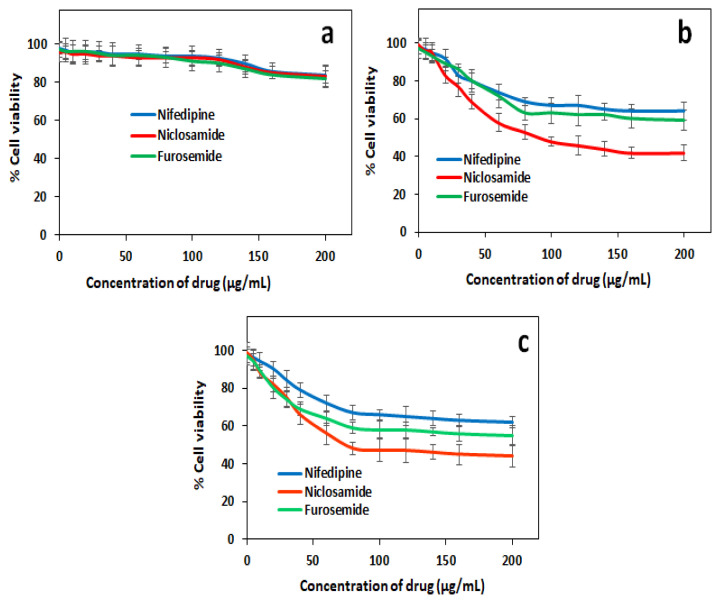
Viability of (a) HEK 293, (b) MCF-7, and (c) MIA PaCa-2 cells exposed to 0–200 μg/mL drugs nifedipine, niclosamide, and furosemide.

The effects of these released drug molecules and hydrogel structure on healthy and cancer cell lines were investigated. Drug concentrations and cell viability measurements were made over time to evaluate the drug release system of the drug-containing hydrogels against HEK-293 (Figure 6a), MCF-7 (Figure 6b), and MIA PaCa-2 (Figure 6c) cells. After reattachment, media of the cells were discarded, and fresh media containing different concentrations of test compounds (0, 5, 10, 20, 40, 60, 80,100, 120, 140, 160, and 200 μg/mL) were added to each well (Figure 6). Samples were prepared as triplicates. Only cell-, only media-containing test compounds for each concentration were used as negative control wells. The drug concentration was determined as 80 μg/mL with the Alamar blue test.

**Figure 7:**
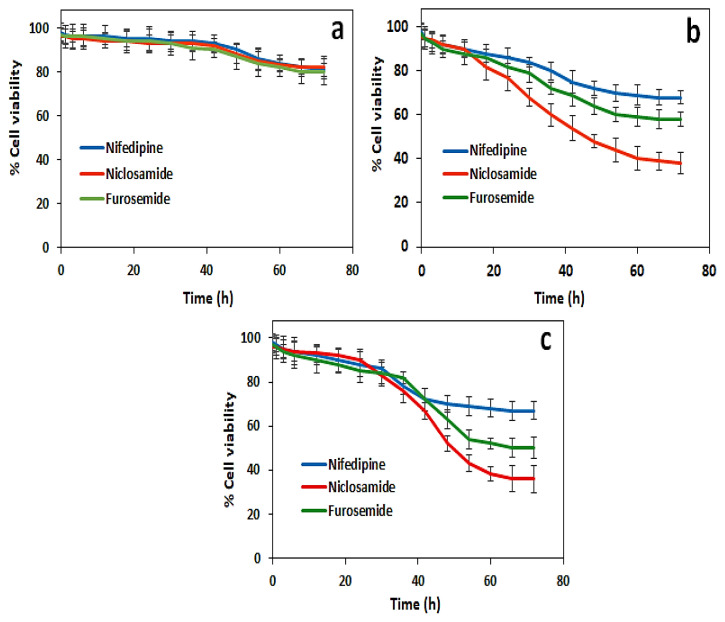
Drug release-induced cell viability by using the Alamar blue assay in (a) HEK 293, (b) MCF-7 cells, and (c) MIA PaCa-2.

In vitro, drug-release studies were tested with drug-loaded hydrogels to obtain results consistent with cumulative release results. In vitro release studies were done on HEK 293, MCF-7, and MIA PaCa-2 cell lines (Figures 7a–7c). The drugs niclosamide, nifedipine, and furosemide caused 62%, 32%, and 42% deaths in MCF-7 cells, respectively. Moreover, niclosamide, nifedipine, and furosemide caused 64%, 33%, and 50% of deaths in MIA PaCa-2, respectively. However, the death rate (around 20%) was less observed in the HEK 293 healthy cell line. Niclosamide has been shown to have more cytotoxic effects on cancer cells than those of furosemide and nifedipine showed.

**Figure 8:**
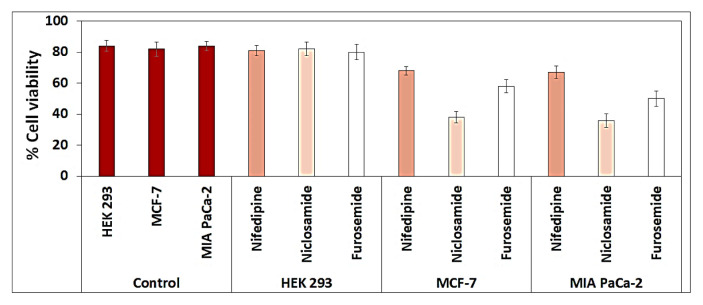
Viability of MCF-7, MIA PaCa-2, and HEK 293 cells exposed to pure HEMA and drug molecule-loaded HEMA for 40 h.

The resulting cytotoxicity test bar chart was shown in Figure 8. Using the results of this cytotoxicity test, IC50 values (semimaximum inhibitory concentrations) were calculated with SigmaPlot 11.0 and are summarized in Table 2.

The Alamar blue experiment was performed using equivalent doses of hydrogels containing the drug molecules. According to in vitro drug release research, the drug release occurred within the first 72 h. The death of MCF-7, MIA PaCa-2, and HEK 293 cells was at the maximum after 40–44 h (Figure 8). However, the lethal effects of drug molecules loaded with hydrogels on HEK-293 were much lower than on MCF-7 and MIA PaCa-2. Application of hydrogel structures containing the niclosamide to cells for 40 h showed inhibition rates of 62% at the MCF-7, 64% at the MIA PaCa-2, and 18% at the HEK-293 cell line. As a result of the release of the furosemide from hydrogel to cells, 42%, 50%, and 20% deaths were observed in MCF-7, MIA PaCa-2, and HEK 293 cell lines, respectively. Hydrogel structures containing nifedipine also showed inhibition rates of 36% in MCF-7, 45% in MIA PaCa-2, and 17% in the HEK-293 cell line. These results showed similarity with the results from the 72-h inhibition test. The results of the release and incubation of niclosamide showed that it has a higher cell death effect than other drug molecules.

**Figure 9:**
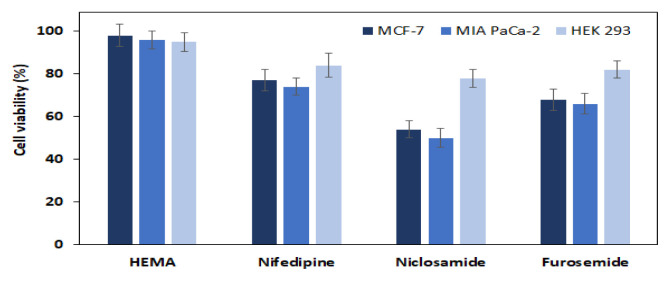
Viability of artificial blood containing cancer cells MCF-7, MIA PaCa-2, and HEK 293 cells exposed to pure HEMA (control) and drug molecule-loaded HEMA for 40 h.

HEMA-based hydrogels were demonstrated to be biocompatible with cells, showing good pharmacological potential. Experiments were conducted on artificial blood samples to support this condition. A safe and effective blood substitute commonly referred to as “artificial blood” but more scientifically referred to as “oxygen therapeutic agent”, has recently given quite a boost to in vivo studies. Many trials are conducted on artificial blood to support in vivo studies and successful results are obtained. In order to support our research, the effects of HEMA hydrogel and drug molecules on artificial blood were investigated. The results obtained were supported by in vitro studies and the results in vivo are summarized in Figure 9. As a result of the release of the niclosamide from hydrogel to artificial blood, 46%, 50%, and 22% deaths were observed in artificial blood containing MCF-7, MIA PaCa-2, and HEK 293 cell lines respectively. Hydrogel structures containing nifedipine also showed inhibition rates of 33% in MCF-7, 36% in MIA PaCa-2, and 16% in the HEK-293 cell line. Furosemide drug demonstrated a death rate of 32% in MCF-7, 34% in MIA PaCa-2, 18% in HEK-293 cell line.

**Figure 10:**
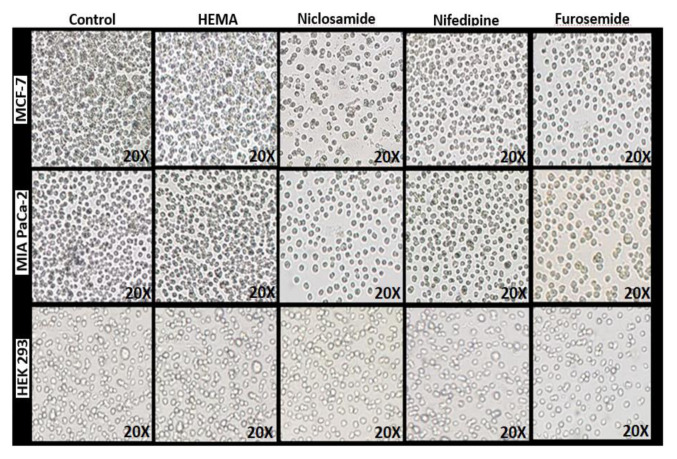
Growth effects of different drug molecules (80 μg/mL) on MCF-7, MIA PaCa-2, and HEK 293 cells after 72-h incubation.

The cytotoxicity tests indicated that all drug molecules had moderately and good cytotoxic activity on the cancer cell lines [51]. The hydrogels containing drug molecules were found to cause more death in cell lines than control and pure hydrogels. Figure 10 showed that the death rate of cancer cells was higher than healthy cells. As a result, niclosamide is the most cytotoxic drug, and furosemide also has moderate cytotoxicity. Furthermore, this study suggests that HEMA hydrogels can be used to preserve drugs and developed as support materials for drug release.

Results of cytotoxicity studies and calculated IC_50_ values were compared. It was observed that the cytotoxicity test results and the calculated IC_50_ values were compatible. From the cytotoxicity tests on cancer cells, it was determined that 80–100 μg/mL drug concentration caused the most death. With this concentration, the maximum cell death was found to be 60%–65% in the presence of a niclosamide drug molecule. With these cytotoxicity test results, the IC_50_ value was calculated as 77–86 μg/mL. When the results are evaluated, the results of the cytotoxicity test and the IC_50_ values (the half maximal inhibitory concentration) agree with each other. The same cytotoxicity tests and IC_50_ values were performed and calculated in HEK 293 healthy cell lines. In HEK 293 cell lines, the maximum mortality rate was observed around 20% by cytotoxicity test. Since the cytotoxicity test result was around 20%, the IC_50_ value could not be defined. These results showed that the cytotoxicity test results and IC50 values gave consistent results.

## 4. Discussion

This study has focused on improving continuous release systems of nifedipine, furosemide, and niclosamide to improve the effectiveness of the drug, which exhibits poor water solubility and poor permeability. The poor solubility of the drug limits its bioavailability and thus greatly reduces its therapeutic efficacy. Hydrogels have been described to improve bioavailability and protection. The cytotoxic effects of drug molecules have been studied in very few studies. However, the controlled release of these drug molecules into cancer cells has been studied for the first time. Compared with other studies, it was determined that the cytotoxic effects of the drug molecules loaded on the HEMA hydrogel were more effective than the pure drug molecules. These anticancer drugs were observed to be effectuated to a large extent by the circulatory system in a published article [9]. In addition to reducing the side effects of drug molecules, their protection with hydrogels has enabled the use of drugs in a healthier way. In many studies, the niclosamide drug molecule has been defined as a more effective anticancer agent than other drug molecules because it has been proven that the drug niclosamide affects more cellular mechanisms than other drug molecules. Niclosamide inhibits Wnt/β-catenin, the mammalian target of rapamycin complex 1, signal transducer and activator of transcription 3 (STAT3), Notch signaling pathways, and nuclear factor-κB, and targets mitochondria in cancer cells to induce cell cycle arrest, growth inhibition, and apoptosis [27]. A number of studies have demonstrated the anticancer activities of niclosamide in vitro and in vivo models. As a result, it can be suggested that HEMA hydrogels prepared within the scope of this study can be drug supplements that can be used in the release studies of nifedipine, furosemide, niclosamide, and similar drug molecules.

## Figures and Tables

**Schema.**
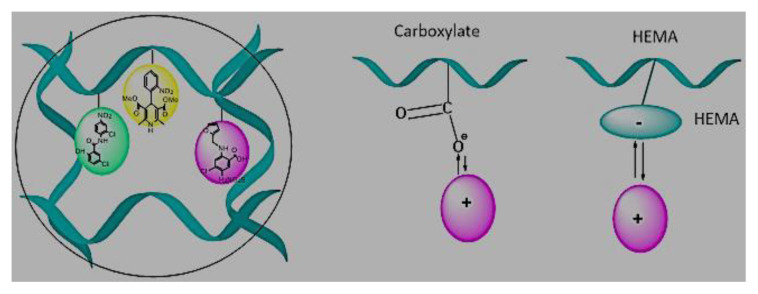
Interaction between drug molecules and hydrogel.

**Table 1:** The hydrogel structure can be studied on different applications as detailed below.

Hydrogel	Structure	Drug	Applications	Reference
N-succinyl chitosan/alginate hydrogel	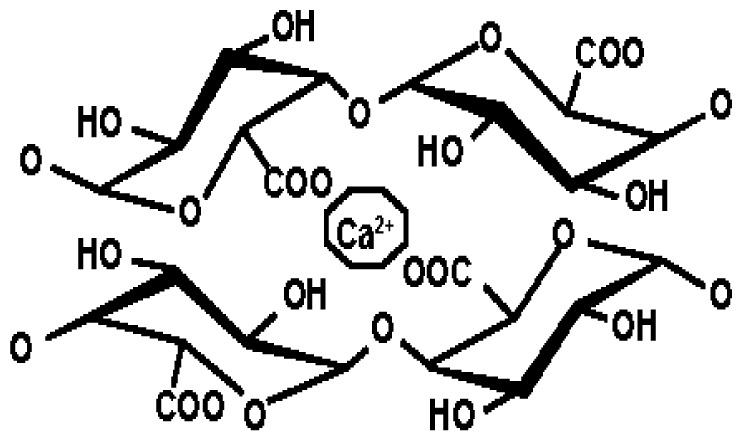	Nifedipine	Controlled delivery	[44]
Pectin-co-poly acrylic acid (Pec-co-poly (AA))	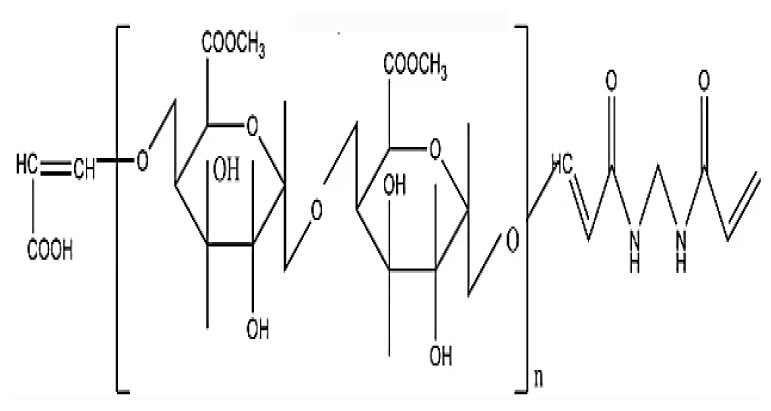	Nifedipine	Controlled delivery	[45]
Hemicellulose-based hydrogels	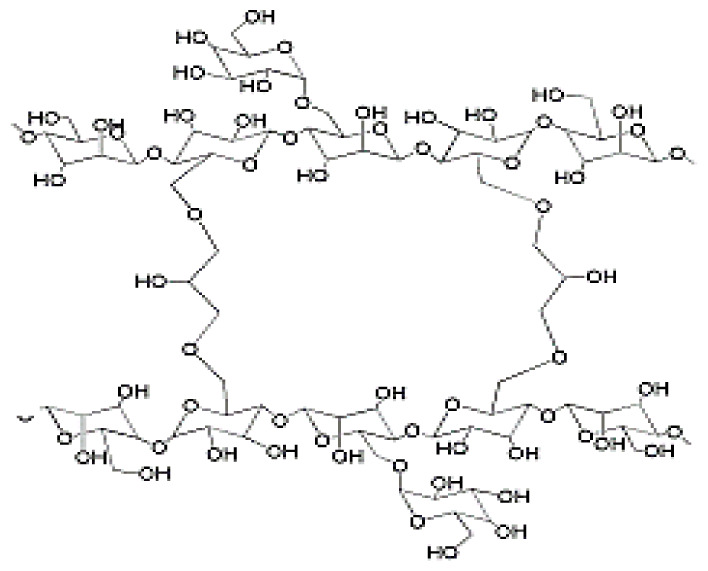	BSA	Magnetically stimulated release	[46]
Alginate bead	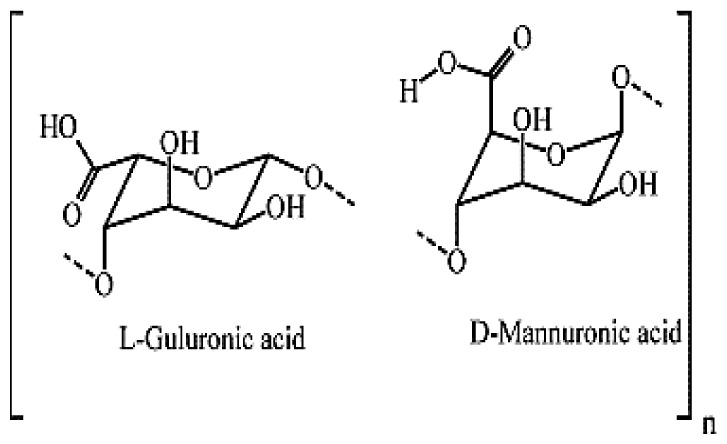	Chlorpheniramine	Drug release	[47]
Chitosan hydrogel	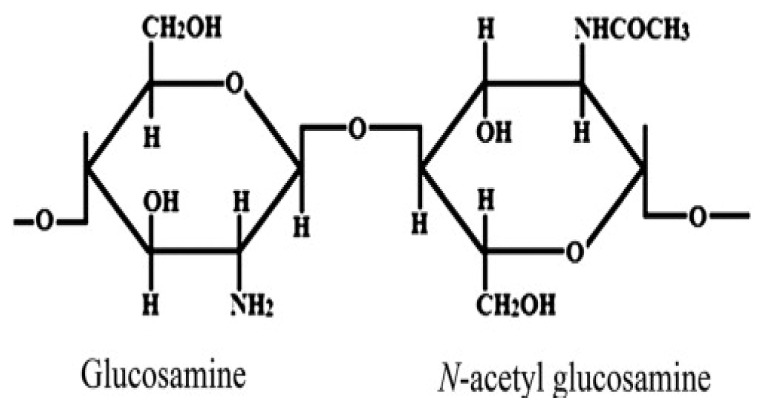	Chlorhexidine	Oral, transdermal, nasal, rectal and ocular drug delivery	[48]
PMAN	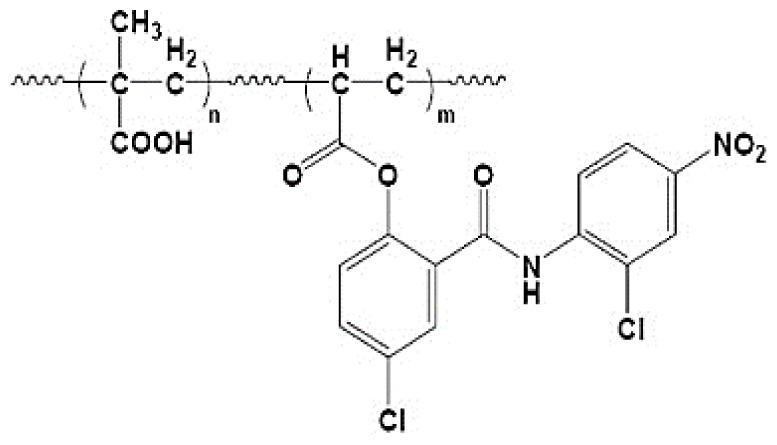	Niclosamide	Solubility and vasoactivity	[49]
HPMC	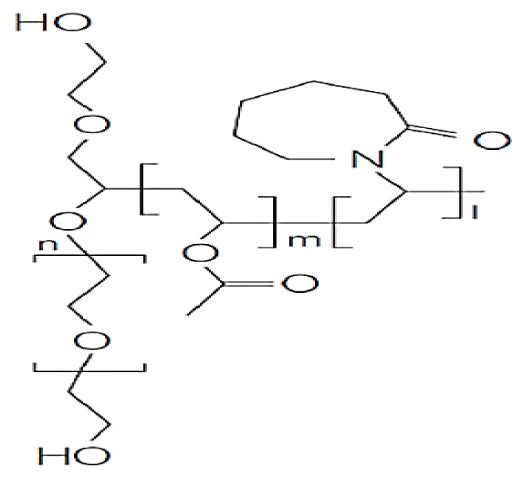	Furosemide	Forming drug solubilizing micelles	[50]
HEMA hydrogel	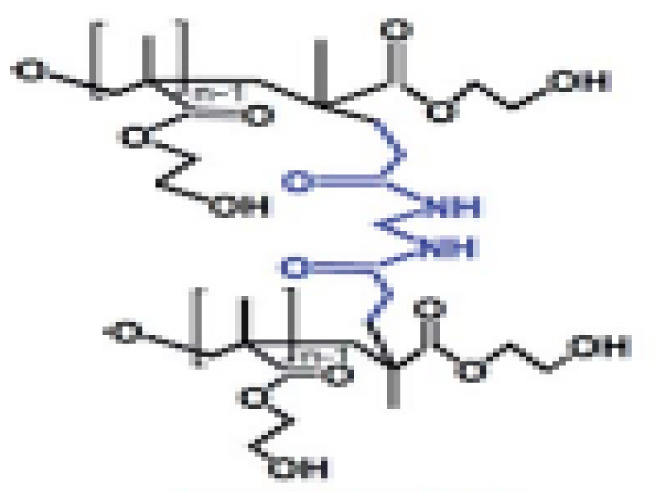	Nifedipine Niclosamide Furosemide	Drug release	is work

**Table 2:** The IC50 of tested drug molecules in different cell lines (MCF-7, MIA PaCa-2, and HEK 293) after 72-h incubation.

Compound	Cytotoxicity (IC50, μM)		
	MCF-7	MIA PaCa-2	HEK 293
Furosemide	95.2 ± 4.7	81.5 ± 5.4	N.D.
Nifedipine	122.3 ± 9.2	118.8 ± 10.6	N.D.
Niclosamide	63.8 ±8.3	66.6 ± 3.9	N.D.
HEMA	N.D.	N.D.	N.D.
Furosemide + HEMA	107.5 ± 7.6	99.1 ± 6.3	N.D.
Nifedipine + HEMA	133.7 ± 9.7	128.5 ± 11.4	N.D.
Niclosamide + HEMA	86.2 ± 10.3	77.3 ± 4.4	N.D.

The data is described as a mean ± SD of three free experimental definitions applied in triplicate

N.D. not detected
